# Mechanochemical Control of Symmetry Breaking in the *Caenorhabditis elegans* Zygote

**DOI:** 10.3389/fcell.2020.619869

**Published:** 2021-01-18

**Authors:** Wan Jun Gan, Fumio Motegi

**Affiliations:** ^1^Temasek Life-Sciences Laboratory, Singapore, Singapore; ^2^Department of Biological Sciences, National University of Singapore, Singapore, Singapore; ^3^Mechanobiology Institute, National University of Singapore, Singapore, Singapore; ^4^Institute of Genetic Medicine, Hokkaido University, Sapporo, Japan

**Keywords:** symmetry breaking, polarization, cortical contractility, Aurora-A, *Caenorhabditis elegans*

## Abstract

Cell polarity is the asymmetric organization of cellular components along defined axes. A key requirement for polarization is the ability of the cell to break symmetry and achieve a spatially biased organization. Despite different triggering cues in various systems, symmetry breaking (SB) usually relies on mechanochemical modulation of the actin cytoskeleton, which allows for advected movement and reorganization of cellular components. Here, the mechanisms underlying SB in *Caenorhabditis elegans* zygote, one of the most popular models to study cell polarity, are reviewed. A zygote initiates SB through the centrosome, which modulates mechanics of the cell cortex to establish advective flow of cortical proteins including the actin cytoskeleton and partitioning defective (PAR) proteins. The chemical signaling underlying centrosomal control of the Aurora A kinase–mediated cascade to convert the organization of the contractile actomyosin network from an apolar to polar state is also discussed.

## Introduction

Multiple tissues are generated during the development of multicellular organisms. The morphogenesis of these tissues is characterized by spatially biased rearrangement of cells along the major body axes. These complex tissues originate from the single-celled zygote, which is formed following fertilization of an oocyte with a sperm. The fertilized zygote initially exhibits no predetermined spatial asymmetry and initiates spatial differences in the shape, structure, and function between the opposite poles. The first step of this process is called symmetry breaking (SB), during which the uniformity of the zygote is disrupted to generate a spatially biased cellular structure. Under most physiological conditions, the zygote responds to local transient “cues” and amplifies microscopic inhomogeneity into stable macroscopic asymmetry. A cue can be a localized landmark either inherited from the oocyte, spontaneously produced by the zygote, or a type of extrinsic stimulus (Li and Bowerman, 2010). The *Caenorhabditis elegans* zygote serves as a useful model organism to understand the mechanism(s) underlying SB. Similar to mammalian oocytes (Santella et al., 1992), the *C. elegans* oocyte is formed without predetermined polarity or extracellular stimuli. Upon entering the reproductive organ known as the spermatheca, the shape of an oocyte is transformed from cuboidal to ovoid (McCarter et al., 1999). A sperm usually enters the oocyte from its leading edge of entering the spermatheca. Shortly after fertilization, the zygote begins to form an extracellular matrix known as eggshell, which provides physical protection to the zygote shape (Olson et al., 2012). The zygote then undergoes polarization, which leads to spatial segregation of the actin cytoskeleton and the presence of conserved signaling proteins in a head-to-tail orientation.

## Mechanochemical Polarization of the *Caenorhabditis elegans* Zygote

**Figure 1:**
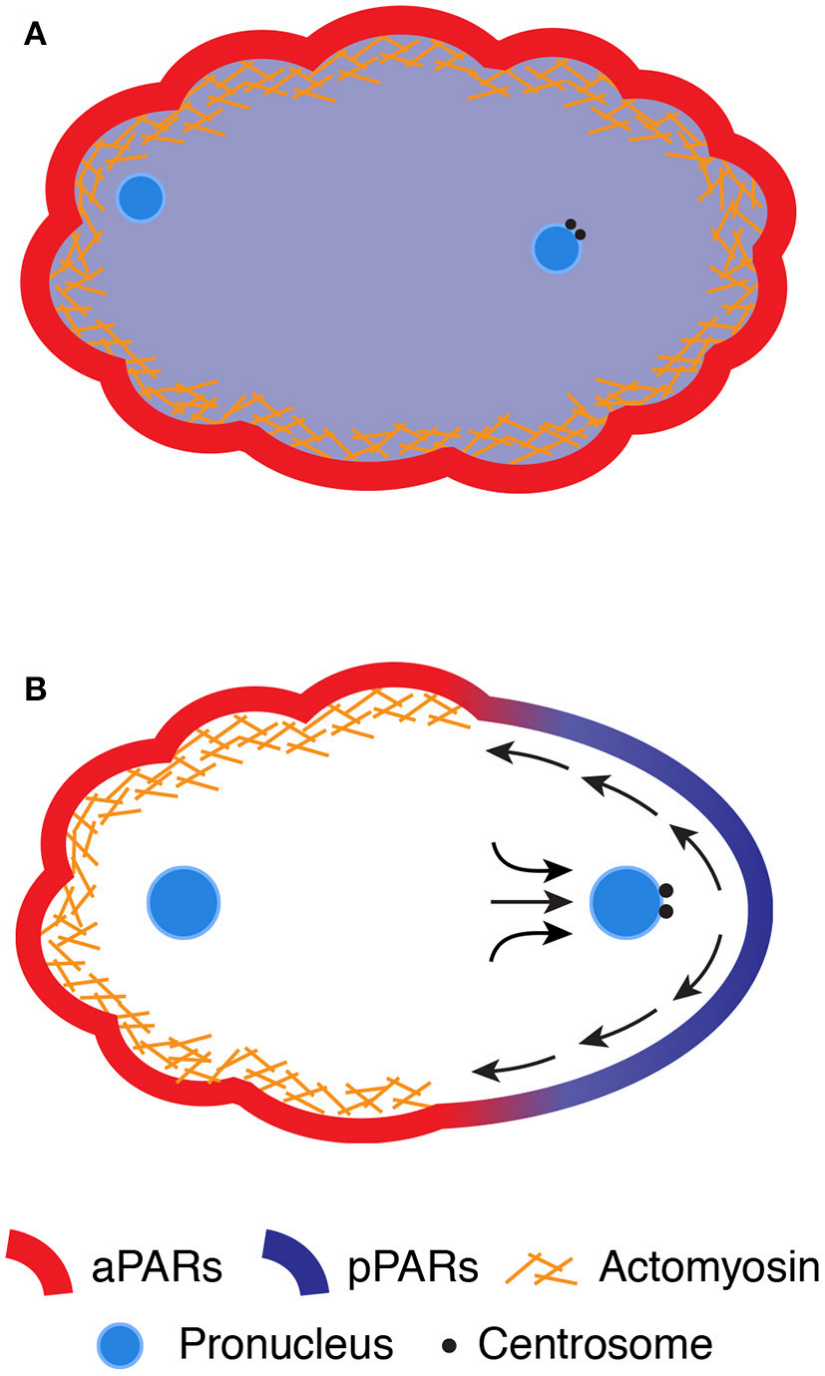
SB in the *C. elegans* zygote. **(A)** The cell cortex enriches of the contractile actomyosin network, which exhibits dynamic cycles of contraction and relaxation, whereas the maternal and paternal chromosomes decondense into pronuclei in the cytoplasm. The aPARs are distributed evenly along the cortex, whereas the pPARs are localized throughout the cytoplasm. **(B)** While the paternal pronucleus approaches the posterior pole, centrosomes send unknown signals to the cortex to initiate SB. The contractile actomyosin network and aPARs are segregated toward the anterior pole via cortical flow, whereas the pPARs are translocated from the cytoplasm to the smoothing posterior pole of the zygote.

Upon entry of the sperm, the zygote undergoes two rounds of meiosis to reduce the number of duplicated maternal chromosomes (Schneider and Bowerman, 2003; Johnston et al., 2006). The excess chromosomes are expelled from the zygote near the pole proximal to the maternal nucleus (Schneider and Bowerman, 2003). Shortly after the completion of meiosis, the zygote starts dynamic cycles of contraction and relaxation of the cell cortex, which is the layer greatly enriched with the actin cytoskeleton underneath the plasma membrane (Figure 1A) (Schneider and Bowerman, 2003; Michaux et al., 2018). The actin cytoskeleton is mainly composed of actin filaments and several actin-binding proteins, including non–muscle myosin II (NMYII). The actin filaments are bundled and cross-linked into a complex network structure at the cortex (termed *cortical actomyosin network*) (Chugh and Paluch, 2018). NMYII is an actin filament–crosslinking protein and acts as a molecular motor driven by adenosine triphosphate (ATP). The catalytic cycles of ATP hydrolysis and the mechanical steps of attachment and detachment of NMYII with cross-linked actin filaments generate contractile forces to its associated structure, thereby inducing contraction and relaxation cycles at the cortex (Hill and Strome, 1988; Chugh and Paluch, 2018; Michaux et al., 2018). The formation and contraction of the cortical network are regulated by Rho-type small GTPase (RHO-1 in *C. elegans* and RhoA in mammals), which hydrolyzes the nucleotide guanosine triphosphate (GTP) to guanosine diphosphate (GDP) (Agarwal and Zaidel-Bar, 2019). RHO-1 is inactive when bound to GDP and active when bound to GTP. RHO-1 is inactivated by stimulating the rate of GTP hydrolysis via GTPase-activating proteins (GAPs), whereas RHO-1 is activated by exchanging GDP with GTP via guanine nucleotide exchange factors (GEFs). Dynamic turnover of RHO-1 between the two states has been correlated with the cyclic contraction and relaxation behaviors of the actomyosin-enriched cortex (Nishikawa et al., 2017; Michaux et al., 2018). The cortex also contains conserved regulators of cell polarity, known as partitioning defective (PAR) proteins. Six PAR proteins (PAR-1 to PAR-6) have been identified in *C. elegans* by genetic screening (Morton et al., 1992, 2002; Levitan et al., 1994; Etemad-Moghadam et al., 1995; Guo and Kemphues, 1995; Watts et al., 1996). Four of the PAR proteins (PAR-1, PAR-2, PAR-3, and PAR-6) are asymmetrically distributed throughout the zygote: PAR-1 and PAR-2 are localized at the posterior cortex, whereas PAR-3 and PAR-6 are enriched at the anterior cortex (Etemad-Moghadam et al., 1995; Guo and Kemphues, 1995; Boyd et al., 1996; Watts et al., 1996). Later studies found that protein kinase C (PKC-3) and cell division control protein 42 homolog (CDC-42) are localized at the anterior cortex (Tabuse et al., 1998; Hung and Kemphues, 1999), and lethal giant larvae-1, LGL-1, and the CDC-42 GAP, CHIN-1, at the posterior cortex (Beatty et al., 2010, 2013; Hoege et al., 2010; Kumfer et al., 2010). PAR-3, PAR-6, PKC-3, and CDC-42 are referred to as anteriorly localizing PAR proteins (aPARs), and PAR-1 and PAR-2 as posteriorly localizing PAR proteins (pPARs). Prior to SB of the zygote, all PAR proteins are symmetrically distributed. PAR-3, PAR-6, and PKC-3 are distributed throughout the cortex, whereas PAR-1, PAR-2, and CHIN-1 are homogeneously present in the cytoplasm (Figure 1A) (Etemad-Moghadam et al., 1995; Guo and Kemphues, 1995; Boyd et al., 1996; Watts et al., 1996; Beatty et al., 2013).

Shortly after meiotic exit, the zygote begins to establish distinct cortical domains with different cortical contractile properties and PAR protein concentrations (Figure 1B). The zygote initially develops actomyosin-based contractility throughout the cortex, which generates forces for the formation of characteristic ruffles on the cell surface (Nishikawa et al., 2017; Michaux et al., 2018). At the onset of polarization (i.e., SB), the zygote locally ceases cortical contraction and forms a smooth-shaped noncontractile cortical domain, which expands from the future posterior pole to half of the cortex. The expansion of the smooth domain coincides with the flow of yolk granules beneath the cortex toward the anterior pole (Hird and White, 1993). The formation of cortical ruffles and the cortical flow can be blocked by the loss of cortical contractility [via inhibition of either NMYII (NMY-2 and myosin light chain-4) (Guo and Kemphues, 1996; Shelton et al., 1999) or the actin polymerizing factor formin (cytokinesis defect-1, CYK-1) (Severson et al., 2002; Hirani et al., 2019)], indicating a critical role of actomyosin-based contractility in this process. These findings imply that contractile asymmetry may induce the establishment of a high-to-low gradient in mechanical tension at the cortex along the anteroposterior axis, which leads to a flow of the actomyosin network toward the anterior pole. However, ultraviolet laser-based ablation experiments revealed the lack of a cortical tension gradient along the anteroposterior axis and instead supported an alternative model in which such contractile asymmetry could mediate asymmetry in hydrodynamic forces within the cortical layer (Kruse et al., 2005; Mayer et al., 2010). A model of hydrodynamic forces depends on the balance of forces applied to the cortex including actomyosin contractile forces and friction caused by compression or expansion of viscoelastic materials on the cortex. Theoretical consideration of the hydrodynamic forces can better explain the flow and density of actomyosin network during polarization (Mayer et al., 2010). However, the physical properties of the cortex, such as hydrodynamic forces, viscosity, and elasticity, have not yet been directly measured during SB.

Simultaneously with the establishment of contractile asymmetry, aPARs (PAR-3, PAR-6, aPKC, and CDC-42) become enriched at the anterior high-contractility domain, whereas pPARs (PAR-1 and PAR-2) translocate from the cytoplasm to the posterior low-contractility domain (Figure 1B). The boundary between the high- and low-contractility domains corresponds precisely with that between the aPAR and pPAR domains (Schenk et al., 2010; Goehring et al., 2011a). Consistent with the tight link between cortical contractility and the PAR domains, live cell imaging experiments revealed the comigratory behaviors of NMYII foci and PAR-6 at the cortex during polarization (Munro et al., 2004). These observations suggest that aPAR is stably embedded within the actomyosin-enriched cortical layer and thus passively transported by the advected flow of the actomyosin network (Munro et al., 2004). This model is also supported by theoretical modeling, which proposes that the velocity of cortical flow is sufficient to passively redistribute freely diffusing aPARs along the anteroposterior axis (Munro et al., 2004). Such advected movement of aPARs depletes aPARs from the posterior pole, allowing pPARs to translocate from the cytoplasm to the posterior domain (Motegi et al., 2011). Once aPARs and pPARs establish cortical asymmetry, both complexes are mutually excluded from the cortex via antagonistic phosphorylation reactions (see the review by Lang and Munro, 2017). Thus, polarization of the *C. elegans* zygote can be understood as a combination of mechanical forces by rearrangement of the cortical actomyosin network with chemical reactions between the aPAR and pPAR complexes.

## Induction of SB by the Centrosome

In parallel, the mechanism employed by the zygote for SB in the actomyosin network was investigated. Most genes that contribute to polarization of the zygote (i.e., PAR genes) were found to carry recessive maternal-effect embryonic-lethal mutations. These maternally provided factors are believed to sense the entry or location of paternally provided factors, as the polarization cascade is initiated by sperm entry (Goldstein and Hird, 1996). A sperm normally enters an oocyte from the side opposing the nucleus. Shortly after the completion of meiosis II, the paternal pronucleus appears near the pole opposite of the maternal pronucleus (Goldstein and Hird, 1996). The paternal pronucleus is associated with the centrosome, an organelle that serves as the main microtubule-organizing center during mitosis. The centrosome is removed from the oocyte, but maintained in sperm and thus is a paternally provided factor in the fertilized zygote (Cowan and Hyman, 2004). The loss of centrosomes, but not paternal chromosomes, during spermatogenesis compromises polarization of the zygote, indicating that the centrosomes and/or associated factors are indispensable for zygote polarization (Sadler and Shakes, 2000). Consistent with the role of centrosomes in polarity induction, SB in cortical contractility will occur from the cortex closest to the position of the centrosomes in the zygote (Bienkowska and Cowan, 2012). Even in mutant zygotes with the centrosomes located near the maternal pronucleus during meiosis, SB is always triggered from the cortex proximally to the centrosomes, which results in the establishment of a reversed pattern of cortical flow and PAR distribution (Goldstein and Hird, 1996; Kimura and Kimura, 2020). These findings indicate that zygote polarization is induced by centrosomes.

Each centrosome contains two centrioles that harbor a matrix of proteins, known as pericentriolar material (PCM) (Chrétien et al., 1997). The PCM then recruits “client” proteins to stimulate nucleation of the microtubules, thereby forming the microtubule-organizing center (Woodruff et al., 2017). This recruitment process, known as centrosome maturation, is generally stimulated during mitosis. Genetic mutations that result in defective centrosome maturation, such as those of the PCM core proteins spindle-defective protein SPD-2 (O'Connell et al., 2000), SPD-5 (Hamill et al., 2002), and the cyclin-dependent kinase 2 (CDK-2)/cyclin E (Cowan and Hyman, 2006) complex, failed to polarize cortical contractility and PAR distribution. Ablation of the centrosomes just prior to the onset of SB also blocked the establishment of asymmetric cortical domains (Cowan and Hyman, 2004). This evidence indicates that the centrosome is the cue triggering SB. Notably, centrosomes distant from the cortex can also induce SB (Bienkowska and Cowan, 2012), suggesting that the “cue” generated by the centrosomes can be transmitted to the cortex through the cytoplasm.

## Centrosomal Aurora A Kinase (AIR-1) Inhibits Cortical Contractility at the Posterior Pole

Three recent studies have proposed that AIR-1 plays a key role in centrosome-mediated SB (Kapoor and Kotak, 2019; Klinkert et al., 2019; Zhao et al., 2019). AIR-1 is a highly conserved serine/threonine kinase that is recruited to the centrosome and stimulates centrosome maturation during mitosis (Schumacher et al., 1998). *C. elegans* zygotes depleted of AIR-1 exhibit defects in the recruitment of PCM, including SPD-2 and SPD-5, microtubule assembly at the centrosomes, and the establishment of cortical polarity (Schumacher et al., 1998). Similar to AIR-1–depleted zygotes, zygotes depleted of either SPD-2 or SPD-5 failed to trigger SB in the actomyosin network (O'Connell et al., 2000; Hamill et al., 2002). AIR-1 is essential to recruit SPD-2 and SPD-5 to centrosomes and vice versa, suggesting that AIR-1, SPD-2, and SPD-5 are engaged with a positive feedback to stimulate centrosome maturation and SB. Such interlinks among the PCM proteins (deletion of one dissociates the assembly of many others) complicate the identification of the specific SB cue conveyed by the PCM.

**Figure 2:**
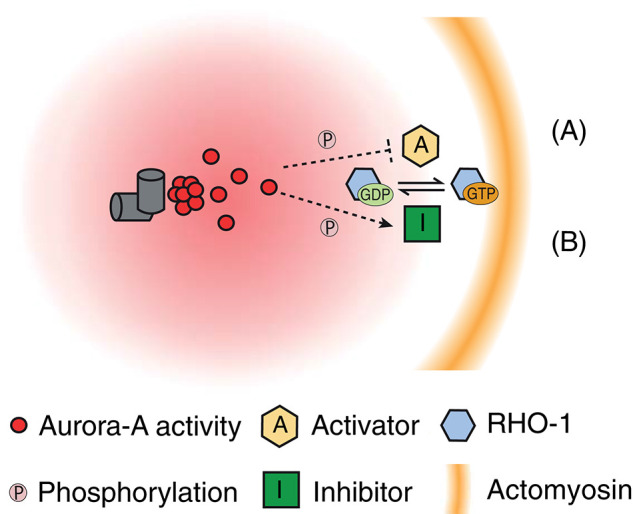
Possible working models of AIR-1 transmission of actomyosin-derived inhibitory signals from the centrosome to the cortex during SB. AIR-1 is speculated to diffuse from the centrosome to the cortex, which may form a gradient of AIR-1 activity centered around the centrosome. **(A)** “Inhibition of RHO-1 activator” model: AIR-1–mediated phosphorylation can directly or indirectly inhibit the activities of RHO-1 activators (such as RhoGEF) at the cortex proximal to the centrosome. **(B)** “Activation of Rho-1 inhibitor” model: AIR-1–mediated phosphorylation can directly or indirectly stimulate the activities of RHO-1 inhibitors (such as RhoGAP) at the cortex proximal to the centrosome.

Three recent studies all demonstrated that AIR-1 is essential for SB in the contractile actomyosin network. A specific role of AIR-1 in reorganization of the actin cytoskeleton was highlighted from the cortical distribution of NMYII in zygotes depleted of AIR-1 after SB (during late prophase) (Kapoor and Kotak, 2019; Klinkert et al., 2019; Zhao et al., 2019). In contrast to control zygotes (such as those depleted of SPD-5), AIR-1–depleted zygotes showed abnormal maintenance of higher activity of RHO-1 (Kapoor and Kotak, 2019; Klinkert et al., 2019; Zhao et al., 2019). This observation implies a unique role of AIR-1 in the inhibition of the cortical actomyosin network, possibly via suppression of RHO-1 activation. Expression of GFP-tagged AIR-1 was able to restore centrosome maturation but unable to rescue SB in zygotes depleted of endogenous AIR-1, highlighting a specific requirement of AIR-1 in SB (Zhao et al., 2019). To support a role of AIR-1 nearby the cortex, Zhao et al. (2019) demonstrated that artificial targeting of AIR-1 to the polar cortex in SB-defective zygotes induced SB in both cortical NMYII and PAR-6. These results delineate a SB cascade in which AIR-1 acts as the centrosomal cue that locally inhibits the actomyosin contractility at the cortex proximal to the centrosome. The downstream signaling of AIR-1 to inhibit formation of a contractile actomyosin network remains unclear. Thus, several models of the inhibition of the formation of a contractile actomyosin network by the transmission of signals facilitated by AIR-1 from the centrosome to the cortex during SB have been proposed (Figure 2).

The first possibility is that AIR-1 diffuses from the centrosome to the cortex and directly phosphorylates a cortical substrate that controls RHO-1 activity. The RhoGEF ECT-2, which activates RHO-1 and stimulates cortical contractility during polarization, is a candidate substrate of AIR-1. ECT-2 contains a lipid-binding motif at the C-terminal region and localizes to the cortex in the *C. elegans* zygote. During SB, ECT-2 is locally excluded from the cortex proximal to the centrosome, but not from the posterior pole of zygotes with nonfunctional centrosomes (i.e., those depleted of either AIR-1 or SPD-5) (Kapoor and Kotak, 2019; Zhao et al., 2019). Kapoor and Kotak (2019) also suggested the serine-rich protein NOP-1, which promotes RHO-1–dependent stimulation of cortical contractility, as another candidate. Indeed, loss of NOP-1 reduced or abolished cortical flow during polarization and prevented a subset of zygotes from establishing polarized PAR domains (Tse et al., 2012). However, at present, there is no direct evidence of interactions among AIR-1, ECT-2, and NOP-1 to confirm these hypotheses.

As a second possibility, AIR-1 could activate a substrate that promotes inactivation of RHO-1. As a potential candidate, RhoGAPs stimulate hydrolysis of the RHO-1 GTPase. Rho-GAP domain-containing protein RGA-3, RGA-4, and cytokinesis defect CYK-4 have been characterized as RhoGAPs that act on RHO-1 in *C. elegans*. RGA-3 and RGA-4 have high similarity and are likely to function in a redundant manner. Codepletion of RGA-3 and RGA-4 causes hyperactivation of RHO-1 and hypercontractility but does not block cortical flow during polarization (Schmutz et al., 2007; Schonegg et al., 2007). In contrast to RGA-3 and RGA-4, CYK-4 is required for reorganization of the actomyosin network during SB and cytokinesis (Jantsch-Plunger et al., 2000; Jenkins et al., 2006). Jenkins et al. showed that depletion of CYK-4 blocked SB in the actomyosin network and cortical PAR distribution in 33–50% of zygotes (Jenkins et al., 2006). Notably, the contribution of CYK-4 to SB is strictly paternal (Jenkins et al., 2006), indicating that CYK-4 provided by sperm triggers SB. Consistent with this paternal contribution, immunostaining revealed that CYK-4 was enriched around the sperm pronucleus (Jenkins et al., 2006). These findings support a model in which CYK-4 acts as a GAP that inactivates RHO-1 at the cortex around the sperm pronucleus during SB. However, this model is contradictory to the findings of several recent studies, which demonstrated that a function of CYK-4 is the local stimulation of RHO-1 activity during cytokinesis. CYK-4 can directly interact with RhoGEF ECT-2 and promote RHO-1 activation at the cleavage furrow during cytokinesis (Zhang and Glotzer, 2015; Gómez-Cavazos et al., 2020). Given that CYK-4 carrying the E448K mutation in the GAP domain has no effect on SB, the GAP activity of CYK-4 might be dispensable during SB (Tse et al., 2012). It is paradoxical that without GAP activity during SB, CYK-4 can serve as an inhibitor of RHO-1, but this does not exclude the possibility that CYK-4 may require other associated proteins or posttranscriptional modifications to sense centrosomal AIR-1 and inhibit cortical contractility. The hypothesis of CYK-4 as downstream target of AIR-1 warrants further investigations.

Although AIR-1 is proposed to diffuse from the centrosome and induce SB at the cortex, the possible involvement of a secondary “messenger” protein that links centrosomal AIR-1 to cortical RHO-1 regulators cannot be ruled out. Microtubules are obvious candidates in this case. Indeed, AIR-1 can indirectly associate with microtubules in a manner dependent on TPXL-1 (*C. elegans* ortholog of targeting protein for *Xenopus* Klp2-like) and colocalize with astral and cortical microtubules (Mangal et al., 2018; Klinkert et al., 2019). However, at present, there is a lack of direct evidence for a role of microtubules in the polarization of the actomyosin networks. Severe depletion of microtubule assembly by RNA interference of tubulin-encoding genes or treatment with nocodazole caused only marginal or no delay on the onset of SB (Strome and Wood, 1983; Cowan and Hyman, 2004; Sonneville and Gönczy, 2004). AIR-1 may also target proteins that control intracellular trafficking linking the centrosome to the cortex. Several proteins involved in intracellular trafficking, such as polarity and osmotic sensitivity defect POD-1 and POD-2, can contribute to SB in the *C. elegans* zygote. POD-1 and POD-2 belong to the coronin family of actin-binding proteins, which connect the actin cytoskeleton to microtubules to regulate intracellular trafficking. Mutations to pod-1 or pod-2 in the zygote result in defective distribution of cortical PARs independent of functions in the regulation of cellular osmolarity (Rappleye et al., 1999; Tagawa et al., 2001).

To understand the complete signaling cascade of centrosome-mediated SB, it is vital to identify potential AIR-1 substrates and other effector proteins. Characterization of this signaling cascade will address several unresolved questions about the mechanism underlying SB, such as “How does the centrosome and centrosome-derived active form of AIR-1 transmit SB signals to the cortex through the cytoplasm?” “How does the centrosome inhibit cortical contractility?” and “Is there any feedback from the actin cytoskeleton to centrosomal AIR-1?” Perhaps unbiased biochemical approaches, such as *in vivo* protein-proximity labeling and comparative mass spectroscopy analysis, can help to further delineate the SB cascade and address these important questions.

## Temporal Control of SB: Suppression of Premature Polarization

SB usually occurs shortly after the completion of meiosis. The timing of SB should be controlled in the zygote because intracellular conditions must be prepared prior to polarization. Wallenfang and Seydoux (2000) reported that PAR proteins can respond to centrosome-independent cues and establish asymmetric cortical domains during meiosis (in zygotes arrested at meiosis). Such meiotic PAR polarization is dependent on microtubules and the formation of meiotic spindles. These observations raised the question as to why the meiotic spindle is unable to trigger polarization under physiological conditions. It has been proposed that premature polarization may be suppressed because of the inefficient ability of the meiotic spindles to induce SB. However, a recent study by Reich et al. (2019) proposed an alternative model in which premature polarization is suppressed by two cell-cycle regulators (i.e., Polo and AIR-1 kinases) during meiosis. Depletion of either Polo or AIR-1 resulted in premature loading of PAR-3 and PAR-6 to the cortex, not only in the fertilized zygote, but also in the oocyte. Given that PAR-3 and PAR-6 can be directly phosphorylated by Polo and AIR-1 in *C. elegans* and *Drosophila melanogaster*, these kinases may directly suppress premature loading of the PAR proteins to the cortex and limit reactivity of the cortex to noncentrosomal SB cues (Wirtz-Peitz et al., 2008; Dickinson et al., 2017). Therefore, these two kinases may perform dual functions in SB: suppression during meiosis and later triggering during mitosis.

However, it remains unclear how the function of these kinases is switched toward SB during oocyte-to-zygote transition, which coincides with the transition from meiosis to mitosis. Activation of the centrosome via centrosome maturation is also suppressed during meiosis but induced during mitosis. It is tempting to speculate that cell cycle–dependent signaling molecules, such as cell cycle–dependent kinases and cyclins, may play direct roles in regulating the activity and/or specificity of Polo and AIR-1. Alternatively, cell cycle–dependent signaling cascades may indirectly control the locations of active Polo and AIR-1 via activation of centrosome maturation. In *C. elegans*, CDK-2 and cyclin E play essential roles in centrosome maturation, as well as SB in zygotes (Cowan and Hyman, 2006). Hence, future investigations of the relationship between CDK-2 and cyclin E, and Polo and AIR-1 kinases are warranted to clarify these hypothetical models and provide a complete view of the cell cycle–dependent control of SB.

## SB Is Facilitated by Multiple Mechanochemical Feedback Cascades

**Figure 3:**
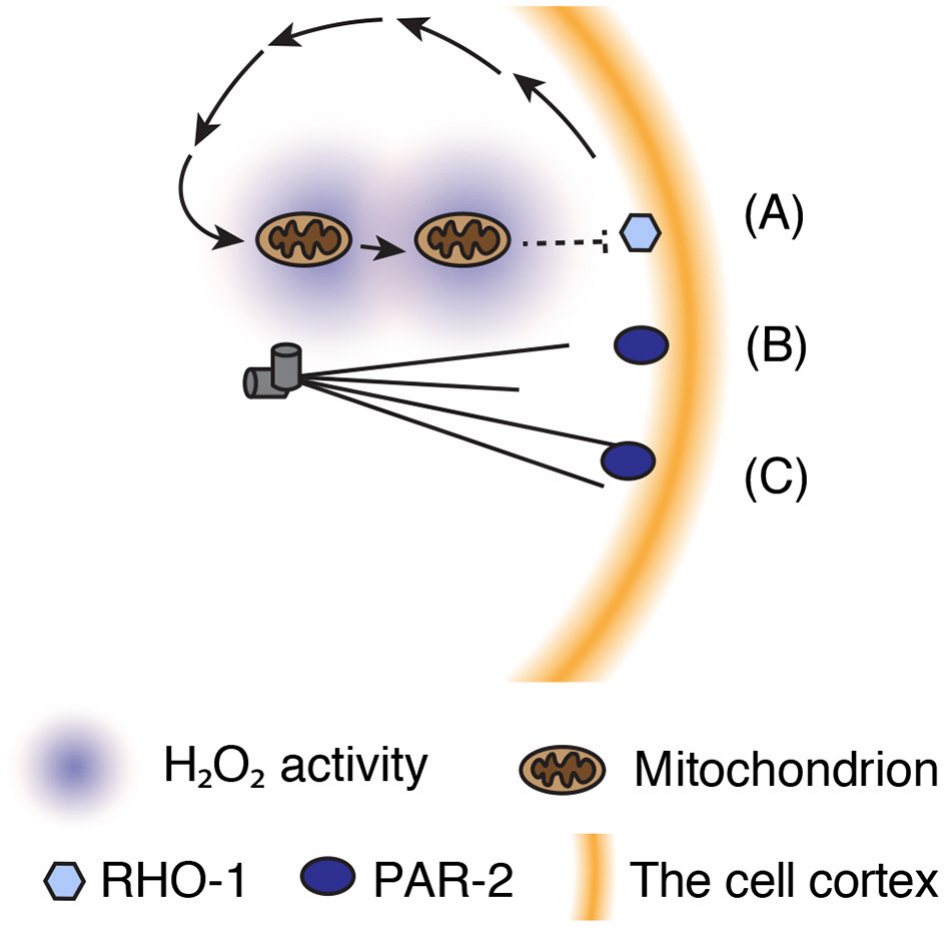
Parallel mechanisms amplify SB to achieve asymmetric patterning of actomyosin and PAR proteins. **(A)** Asymmetric distribution of mitochondria in the cytosol generates H_2_O_2_ gradient, which may oxidize RHO-1 to inhibit its activity. **(B)** Microtubule binding regulates PAR-2 localization onto the posterior domain. **(C)** Membrane curvature facilitates PAR-2 cortical localization and concentration at the posterior pole.

In most SB events, the cell responds to certain cues and amplifies initial transient bias into more stable cellular-scale asymmetry. Recent studies of *C. elegans* zygotes have highlighted several mechanochemical feedback cascades that amplify SB initiated by the centrosome and facilitate stable patterning of the actin cytoskeleton and PAR proteins in the cortex (Figure 3).

Centrosome-mediated SB establishes directed (posterior-to-anterior) flow of cortical proteins. Given that the cortex and cytoplasm are physically connected, a physical force that drives cortical flow creates a hydrodynamic force that moves the cytoplasm in an opposite (anterior-to-posterior) direction. Although the physiological function of cytoplasmic flow in cell polarization remains unclear, a recent study proposed that cytoplasmic flow and intracellular hydrogen peroxide (H_2_O_2_) facilitate polarization of cortical contractility (De Henau et al., 2020). H_2_O_2_ is a reactive oxygen species that is predominantly produced by the mitochondrial electron transport chain and implicated as a transmitter of redox signals. In the *C. elegans* zygote, the cytoplasmic distribution of mitochondria undergoes dynamic changes from a uniform to posteriorly enriched pattern as cortical and cytoplasmic flows emerge during SB (De Henau et al., 2020). Measurements of intracellular H_2_O_2_ levels with a biosensor revealed local enrichment of H_2_O_2_ at the posterior pole during SB (De Henau et al., 2020). The gradient of H_2_O_2_ is built up from an actomyosin-dependent flow that redistributes cytoplasmic mitochondria toward the posterior pole (De Henau et al., 2020). Lowering H_2_O_2_ production by either the paternal or maternal mitochondria delays the timing of SB (De Henau et al., 2020), indicating a role of H_2_O_2_ in ensuring efficient SB. Remarkably, optogenetic manipulation of mitochondria localization toward the pole opposite to the centrosome (in an SB-defective zygote) reversed the pattern of polarization (De Henau et al., 2020). These findings support a model in which H_2_O_2_ contributes to SB either downstream or in parallel with the centrosomal AIR-1–mediated cascade. However, it remains unclear how H_2_O_2_ can inhibit the RHO-1–mediated cortical contraction cascade. RHO-1 itself can be a downstream target of redox signaling, as RHO-1 has two highly conserved cysteine residues for oxidation (Heo and Campbell, 2005; De Henau et al., 2020). Hence, further studies are needed to clarify the target molecule(s) of redox signals and possible relationships with the centrosomal AIR-1–mediated cascade.

Despite the role of AIR-1 in mediating SB in cortical contractility, PAR-2 can be translocated to the polar cortex during mitosis even in zygotes depleted of AIR-1 (Kapoor and Kotak, 2019; Klinkert et al., 2019; Zhao et al., 2019). In AIR-1–depleted zygotes, PAR-2 accumulates at the anterior pole or both poles, but at a significantly slower rate than that of wild-type zygotes. Recruitment of PAR-2 to the polar cortex in the absence of a functional centrosome can be mediated via microtubule-containing structures, such as the astral microtubules of meiotic spindles and meiotic spindle remnants at the anterior pole (Wallenfang and Seydoux, 2000). Binding to microtubules protects PAR-2 from PKC-3-mediated phosphorylation and exclusion from the cortex, leading to the stabilization of cortical PAR-2 (Motegi et al., 2011). Once translocated to the cortex, PAR-2 recruits PAR-1 kinase, while excluding anterior PAR proteins from the cortex, in order to establish a low-contractility cortical domain (Ramanujam et al., 2018).

Recruitment of PAR-2 to the polar cortex in the absence of functional centrosomes can also be stimulated by the macroscopic geometry of the cell, particularly the curvature of the plasma membrane. In zygotes with nonfunctional centrosomes, PAR-2 tends to appear at the high-curvature polar cortex. To test the hypothesis that PAR-2 preferentially associates with the high-curvature cortex, Klinkert et al. (2019) squeezed zygotes into triangular microwells and sharpened the curved regions of the cortex. Under wild-type conditions, PAR-2 was localized along one side of the cortex in half of the zygotes and was distributed at one of the triangular corners in the other half (Klinkert et al., 2019). In contrast, AIR-1–depleted zygotes preferentially positioned PAR-2 at the corner with the highest curvature (Klinkert et al., 2019). This behavior of PAR-2 was independent of the functions of centrosomes and microtubules, indicating that the curvature of the plasma membrane can be viewed as a novel factor that contributes to polarity establishment even in the absence of centrosomes and microtubules. PAR-2 has a phosphoinositide-binding domain and can be oligomerized at the cortex. Similar to proteins containing a bin-amphiphysin-rvs (BAR) domain, the oligomerized form of PAR-2 may form a “bended” shape that preferentially adopts to a plasma membrane with high curvature (Ford et al., 2002; Peter et al., 2004; Miller et al., 2015; Zeno et al., 2018). PAR-2 may sense the specific composition and/or density of phosphoinositides that accumulate at the high-curvature cortex. Hence, it would be informative to elucidate the structural features of oligomerized PAR-2 and reconstruct the curvature-sensing capability of PAR-2 with the use of synthetic liposomes.

## Ensuring the Position of the Centrosome for Efficient SB

**Figure 4:**
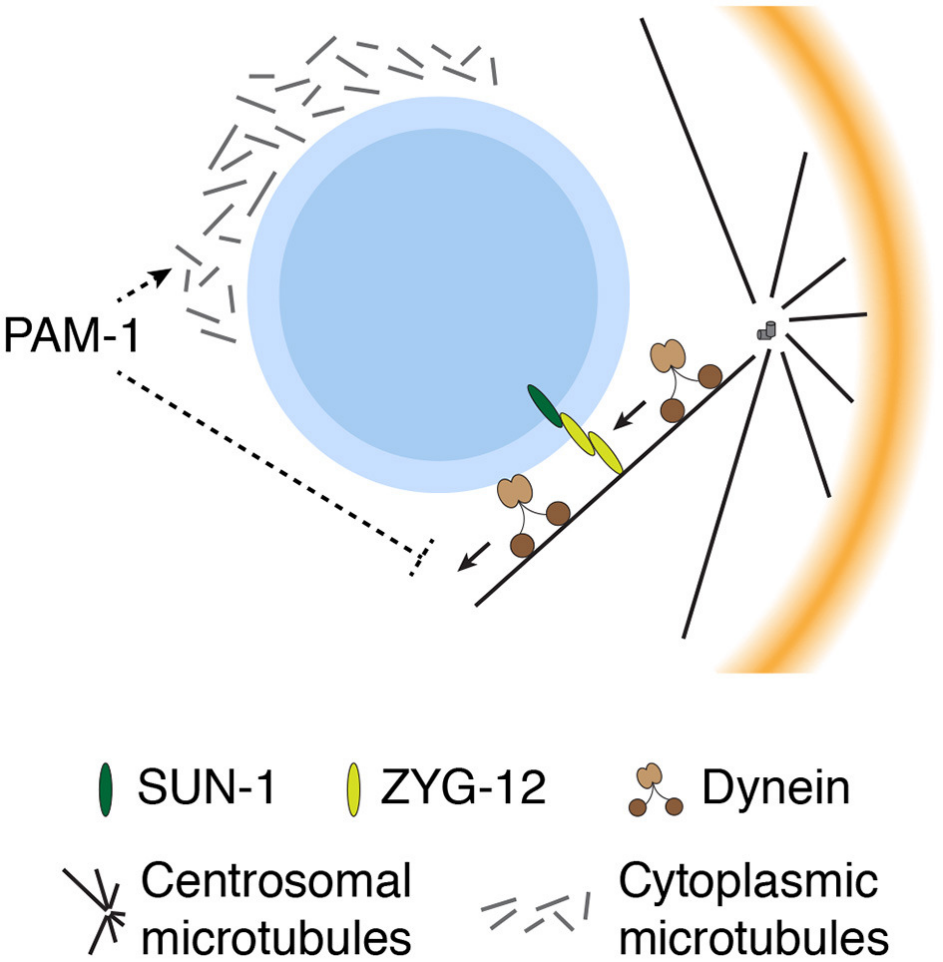
Multiple mechanisms constrain movement of the pronucleus–centrosome complex near the cortex for efficient SB. Cytoplasmic microtubules prevent premature movement of the pronucleus–centrosome complex away from the cortex, whereas ZYG-12 physically links the centrosomes to the pronucleus. PAM-1 may contribute to the formation of cytoplasmic microtubules or suppress dynein-dependent movement of the pronucleus–centrosome complex prior to SB.

In addition to the aforementioned mechanisms that initiate and facilitate SB, the *C. elegans* zygote utilizes other mechanisms to maintain a complex consisting of the paternal pronucleus and centrosomes closer to the cortex prior to SB (Figure 4). Several studies have demonstrated that premature movement and mispositioning of the centrosomes away from the cortex can disrupt polarity, indicating that the proximity between the centrosomes and cortex is critical for efficient SB. This concept was first proposed from genetic studies of *pam-1* mutant zygotes, which exhibited both centrosome mispositioning and defects in the onset of SB (Lyczak et al., 2006; Fortin et al., 2010). Although the *pam-1* mutant zygotes exhibited pleiotropic phenotypes including prolonged meiotic exit, the defect in SB in *pam-1* mutant zygotes was rescued when the position of centrosomes was restricted near the posterior cortex (via inhibition of dynein) (Fortin et al., 2010). This finding suggests a role of PAM-1 in the control of SB through suppression of premature movement of the centrosomes away from the cortex. However, the molecular details of how PAM-1 prevents premature movement of the centrosome are currently unclear.

The second line of evidence was obtained from investigations of centrosome positioning in mutant zygotes with improperly assembled cytoplasmic microtubules. The loss of either γ-tubulin, a tubulin subtype that contributes to nucleation of microtubules at the microtubule minus end, or Ran GTPase dramatically reduced the assembly of microtubules in the cytoplasm (Bienkowska and Cowan, 2012). Reduction in the density of cytoplasmic microtubules inhibits cortically biased movement of the centrosome, resulting in an increased distance from the centrosome to the cortex (Bienkowska and Cowan, 2012). In γ-tubulin–depleted zygotes, the proximity between the centrosome and cortex is closely correlated to the time required for SB, indicating that the efficiency of SB is ensured through constraining centrosome movement by cytoplasmic microtubules.

In the *C. elegans* embryo, the position of the centrosome is also influenced by physical attachment to the nuclear envelope, which is mediated by a member of the hook protein family, zygote defective protein 12 (ZYG-12) (Malone et al., 2003). ZYG-12 is expressed as three distinct isoforms: A, B, and C. ZYG-12 A is associated with the centrosome, in a microtubule-dependent manner, whereas ZYG-12 B and C are enriched in the nuclear envelope, in a SUN-1 protein-dependent manner. The self-binding of ZYG-12 links centrosomal ZYG-12 A to nuclear ZYG-12 B and C (Malone et al., 2003; Minn et al., 2009). In ZYG-12–depleted zygotes, the centrosome is physically separated from the paternal pronucleus and positioned away from the cortex (Malone et al., 2003). Although cortical polarity can be established during mitosis in *zyg-12* mutant zygotes, the kinetics and efficiency of SB have not been investigated.

The position of the centrosome prior to SB is also influenced by cytoplasmic streaming during meiosis. From fertilization to SB, circular cytoplasmic streaming facilitates uncontrolled drifting of the paternal centrosome–pronucleus complex, which occurs orthogonally to the anteroposterior axis in a manner dependent on microtubules and the microtubule motor protein kinesin-1 (Kimura et al., 2017). During cytoplasmic streaming, kinesin-1 transports a network of endoplasmic reticula toward the plus end of the microtubule (Kimura et al., 2017). If by chance cytoplasmic streaming moves the centrosome–pronucleus complex away from the sperm-entry site toward the opposite pole, where the maternal pronucleus is generally located, the centrosome can induce SB at the cortex opposite to the sperm-entry site (Kimura and Kimura, 2020). These observations confirmed that the position of the centrosome at the time of SB, but not the sperm-entry site, specifies the anteroposterior axis.

## Conclusion and Perspectives

Overall, studies of the *C. elegans* zygote have revealed the molecular mechanisms that control SB and the establishment of cell polarity, which include PAR proteins and their regulators. The versatility of the PAR cascade in cell polarization reflects a conserved mechanism that orchestrates the establishment of distinct cortical domains in many types of polarized cells. In addition to PAR proteins, the actin cytoskeleton and RhoA GTPase play crucial roles in SB. The cortical actomyosin network is engaged with cycles of contraction and relaxation before SB, but the cortical PAR proteins remain distributed throughout the cortex, indicating the robustness of the apolar state to prevent random polarization. A recent study reported the involvement of the Polo and AIR-1 kinases in suppression of premature polarization. However, a key to understand this process is whether these kinases have any influence on either the PAR proteins or actin cytoskeleton. Similar to other types of polarized cells, such as neurons and immune cells, SB in the *C. elegans* zygote relies on a localized signaling center that transmits “symmetry breaking signal(s)” to induce local changes in the organization of the cortical actomyosin network. Recent studies identified AIR-1 as the long-sought-after “cue,” which is concentrated by the centrosome to inhibit cortical contractility. Given that activated AIR-1 should trigger SB, it is important to identify the associated activators and substrates. Of equal interest is how the function of AIR-1 switches from prevention of premature polarization to induction of SB during oocyte-to-zygote transition. Recent studies have reported mechanochemical coupling between cytoplasmic flow and redox signaling and that recruitment of PAR-2 to the polar cortex is dependent on the curvature of the plasma membrane. Further investigations will undoubtedly shed light on the molecular basis of these positive feedback loops.

The *C. elegans* zygote is so far the most well-studied system to investigate the intrinsic pathways in SB. In contrast, other model systems including mammalian embryos have highlighted distinct intrinsic pathways that facilitate efficient SB. Mouse preimplantation embryo becomes polarized by segregating PAR proteins along inside–outside orientation at the 8-cell stage. A single cell isolated from the 8-cell stage embryo is capable of polarizing actomyosin contractility and PAR proteins at the cortex in a cell-autonomous manner (Anani et al., 2014; Maître et al., 2016). Asymmetry in cortical contractility in turn directs internalization of a lower-contractile cell in the 16-cell stage embryo (Maître et al., 2016). How SB is induced in the 8-cell stage embryo remains not fully understood. Distinct from *C. elegans* zygote, the cells of the mouse embryo lack centrosomes, suggesting an involvement of other intracellular structures in SB. Interestingly, these cells maintain their cytokinetic bridge structure (also known as midbody) even after cell division and position them toward the outer side of the cortex (Zenker et al., 2017). This bridge structure serves as scaffold for the accumulation of microtubule-associated proteins such as CAMSAP3, thereby acting as a noncentrosomal microtubule-organizing center during interphase (Zenker et al., 2017). The volume of the bridge structure is asymmetric between two daughter cells, resulting in asymmetry in transport of key proteins required for cell polarity to the outer cortex (Zenker et al., 2017). It remains unknown how the bridge structure acquires asymmetric properties after cell division. Recent study of *C. elegans* gastrulation revealed a role of Aurora-B kinase in polarizing the midbody toward the apical cortex and establishing the apical cortical domain (Bai et al., 2020). These findings suggest that mouse embryo may utilize the bridge structure as an intracellular signaling center for cell polarity kinase(s). Recent study also highlighted a role of intermediate filaments including keratins in the induction of cell polarity in mouse embryos (Lim et al., 2020). Keratins function as asymmetrically inherited factors and stabilize the outer cortical domain via interaction with the actin cortex. Asymmetric inheritance of keratins to the outer cells requires the outer cortical domain proteins such as PARD6B and the actin cytoskeleton (Lim et al., 2020), suggesting that keratins may be involved in a positive feedback with these proteins and otherwise may not be the most upstream factor in the SB cascade. SB in mouse embryo is also ensured by extrinsic stimuli including cadherin-based adhesions (Shirayoshi et al., 1983) and mechanical forces through physical attachment with neighboring cells (Samarage et al., 2015; Korotkevich et al., 2017). Therefore, distinct from *C. elegans* zygote, mammalian embryos utilize a combination of intrinsic and extrinsic pathways to ensure efficient SB during embryogenesis.

Understanding the mechanism underlying cell polarization has been a long-standing problem in cell and developmental biology. Particularly, our knowledge of SB induction is far less comprehensive. Recent advancement in imaging technologies and artificial manipulation of cellular signaling has revealed novel mechanistic insights into SB. It is reasonable to employ computer-assisted approaches to achieve quantitative explanations from these insights. Several mathematical models have already contributed to our understanding of the basic principles in *C. elegans* SB (Tostevin and Howard, 2008; Dawes and Munro, 2011; Goehring et al., 2011b; Kravtsova and Dawes, 2014; Gross et al., 2019; Geßele et al., 2020). Further comprehensive assessment and modeling of the concentration, diffusion, and complex composition of the actin cytoskeleton and PAR proteins will be useful to decipher the complex molecular behaviors into simple principles that govern the spatial organization of a cell. In conclusion, utilization of the *C. elegans* zygote as a model to study the mechanochemical reactions underlying SB and polarization has yielded valuable insights into the biophysical basis of the signaling cascades and has advanced the discovery of novel functions of proteins in various developmental contexts.

## Author Contributions

WG and FM prepared, wrote, and revised the manuscript together. All authors contributed to the article and approved the submitted version.

## Conflict of Interest

The authors declare that the research was conducted in the absence of any commercial or financial relationships that could be construed as a potential conflict of interest.
